# ﻿Checklist and keys to Deltocephalinae leafhoppers (Hemiptera, Cicadellidae) from Pakistan

**DOI:** 10.3897/zookeys.1078.47616

**Published:** 2021-12-21

**Authors:** Hassan Naveed, Bismillah Shah, Bilal Saeed Khan, Chengquan Cao, Mick Webb, Yalin Zhang

**Affiliations:** 1 College of Life Science, Leshan Normal University, Leshan, Sichuan 614004, China Leshan Normal University Leshan China; 2 Key Laboratory of Plant Protection Resources and Pest Management of the Ministry of Education, Entomological Museum, Northwest A&F University, Yangling, Shaanxi Province 712100, China Northwest A&F University Yangling China; 3 School of Plant Protection, Anhui Agricultural University, Changjiang West Road 130, Hefei, 230036 Anhui, China Anhui Agricultural University Hefei China; 4 Department of Entomology, Faculty of Agriculture, University of Faisalabad, Punjab 38040, Pakistan University of Faisalabad Faisalabad Pakistan; 5 Department of Life Sciences, Natural History Museum, Cromwell Road, London SW7 5BD, UK Natural History Museum London United Kingdom

**Keywords:** Auchenorrhyncha, distribution, key, morphology, synonyms

## Abstract

Keys to all levels of the subfamily Deltocephalinae (Hemiptera: Cicadellidae) of Pakistan are provided based on published records and original data from recent research. Checklists to the genera and species of Deltocephalinae are also given. A total of 49 genera with more than 100 species are now known from Pakistan. Two new synonyms are proposed, i.e., *Cicadulinastriata* Ahmed, 1986 a junior synonym of *Cicadulinachinai* Ghauri, 1965, **syn. nov.** and *Macrostelesparafalcatus* Naveed & Zhang, 2018 a new junior synonym of *Macrostelesindrina* (Pruthi, 1930), **syn. nov.**

## ﻿Introduction

Cicadellidae, the largest family of Hemiptera, comprises 26–40 subfamilies (depending on the classification used, e.g., Dietrich 2005 and Oman et al. 1990, respectively). Included are nearly 22,000 species of which more than 200 species are known from Pakistan (Khatri and Webb 2010). The largest leafhopper subfamily, Deltocephalinae, is found in all geographical regions and comprises more than 38 tribes and 923 genera (Zahniser and Dietrich 2013). The earliest Deltocephalinae to be recorded from Pakistan were by Pruthi (1930, 1936) who recorded several species from Indian localities which are now in Pakistan, e.g., Lyallpur, Changla Gali and Murree Hills. Thirty-one genera and 57 species of the subfamily were recorded from Pakistan by Khatri and Webb (2010); these authors also provided a checklist to Pakistan Deltocephalinae and illustrated the species, some new. Subsequently, Khatri and Rustamani (2011) provided a key to tribes and genera known at that time from Pakistan and, due to the revised classification of Zahniser and Dietrich (2013), some genera have been transferred from one tribe to another (see Remarks under Deltocephalinae). In this paper we add a further 18 genera and 51 species records, provide checklists and keys to species and include two new species synonymies; a total of 49 genera with more than 100 species is now known from Pakistan.

Much taxonomic work needs to be done for the fauna of Cicadellidae in various countries and this is particularly true for Pakistan. Such studies are not only important to discover the leafhopper diversity but also for pest management in agriculture and forestry as leafhoppers being one of the most important groups of vectors of plant pathogens (Claridge and Wilson 1991; Wilson and Turner 2010).

## ﻿Materials and methods

All specimens were examined with a Leica ZOOM2000 stereomicroscope. Drawings were made using an Olympus drawing tube. Photos were taken by a ZEISS SteREO Discovery.V20 stereomicroscope equipped with a ZEISS AxiocamICc 5 camera that also provided measurements. Adobe Photoshop CS was used to compile photographs. Specimens from Pakistan are deposited in the various collections as indicated in the published records and additional specimens, examined and figured for this study, are deposited in the Entomological Museum, Northwest A&F University, Yangling, Shaanxi, China.

## ﻿Taxonomy

### ﻿Deltocephalinae Fieber

The subfamily Deltocephalinae includes small-to-large, mainly wedge-shaped leafhoppers diagnosed as follows: head with ocelli on anterior margin near to eyes; frontoclypeus not swollen, carinae on anterior margin of head usually absent; lateral frontal sutures reaching to ocelli; antennal ledges reduced or absent; gena large, usually covering proepisternum, with a fine erect seta laterad of lateral frontal suture. Forewing macropterous to brachypterous; if macropterous, with apices usually overlapping at rest (except *Gurawa*); with two or three anteapical cells and often with one or more crossveins between A1 and claval suture; inner apical cell narrowed distally, not reaching to wing apex. Profemur AM1 seta distinct; row AV with short stout setae extending from base to 1/2–2/3 length of femur; intercalary row with various thin setae arranged in one row. Mesotrochanter with apical posteroventral stout seta. Metafemur macrosetal formula usually 2+2+1 with penultimate pair close-set. Metatibia usually anteroposteriorly compressed, ventrally with a median ridge. Male pygofer usually with a membranous cleft at basolateral margin. Valve produced posteriorly, lateral margins short, articulated with pygofer laterally. Subgenital plates articulated with each other and with valve rarely fused to each other and valve (*Goniagnathus*); usually triangular, normally somewhat flattened; with dorsal slot or fold articulating with style. Connective Y-shaped or linear, rarely T-shaped; devoid of anteromedial lobe or process. Style broad at base, bilobed basally; apophysis not elongate. First valvula convex to relatively straight; dorsal sculpturing pattern reaching the dorsal margin or not; sculpturing pattern striate, concatenate, reticulate, imbricate, maculate, or granulose. Second valvula with basal fused section as long as distal paired blades or longer; median dorsal tooth present or not; usually with small to large, regularly or irregularly shaped dorsoapical teeth on apical 1/3 or more; teeth sometimes restricted to apical 1/4, or absent.

Remarks. We treat Deltocephalinae here in its wider sense, following Zahniser and Dietrich (2013) to include Selenocephalini, Mukariini and Penthimiini. We also follow Zahniser and Dietrich (2013) for the placement of genera in tribes; this has particular implications for *Bampurius* placed in Athysanini by Khatri and Webb (2010), here placed in Scaphoideini and the genera placed in Scaphytopiini by Khatri and Webb (2010), i.e., *Grammacephalus* placed here in Scaphoideini, *Masiripius* placed here in Opsiini and *Varta* placed here in Vartiini.

### ﻿Key to tribes and genera of Deltocephalinae from Pakistan

If genera are represented by a single species in Pakistan the species name is given.

**Table:** 

1	Crown with transverse striations or carinae on anterior margin	**2**
–	Crown with anterior margin smooth or shagreen	**9**
2	Clypellus narrow, extending beyond margin of genae, tapered towards apex	**Koebiliini (Grypotina) 3**
–	Clypellus broader, not extending beyond margin of genae	**4**
3	Crown medially longer than next to eyes; aedeagus simple, without processes	***Sohiponawebbi*** (p. 161)
–	Crown with uniform length; aedeagus with lateral processes	***Pinoponaminuta*** (p. 161)
4	Antennae arising near upper corner of eyes	**Drabescini 5**
–	Antennae arising distinctly below upper corner of eyes	**6**
5	Dark robust species; crown similar in length throughout width (Fig. 1); antennal ledges strong; antennae similar in width to head; forewing appendix broad	**Drabescina** (***Drabescusangulatus*)** (p. 156)
–	Pale narrow species; crown distinctly longer medially than next to eyes; antennal ledges weak or absent; antennae much longer than width of head; forewing appendix narrow	**Paraboloponina** (***Dryadomorphapallida*)** (p. 157)
6	Crown slightly longer medially than next to eye	**Athysanini (in part) *Tambocerusbulbulus*** (p. 143)
–	Crown distinctly longer medially than next to eye	**7**
7	Head depressed anteriorly, if not depressed then ocelli on crown close to foremargin; forewing venation reticulate (Fig. 2); aedeagus with single shaft	**Penthimiini 8**
–	Head not so depressed, ocelli on anterior margin; forewing venation not reticulate; aedeagus with two shafts	**Mukariini** (***Mukariasplendida***) (p. 165)
8	Ocelli on anterior margin of crown	***Neodartusacocephaloides*** (p. 170)
–	Ocelli on crown near anterior margin	***Penthimiacompacta*** (p. 170)
9	Robust and squat species (Fig. 3); forewing with appendix extending aroundwing apex (Fig. 57); subgenital plates fused to each other and to valve; connective fused with aedeagus (Fig. 41)	**Goniagnathini (*Goniagnathus*)**
–	Without this combination of characters	**10**
10	Crown produced, pointed anteriorly; genae visible behind eyes in dorsal view; forewing truncate apically	**Vartini (*Vartarubrofasciata*)** (p. 175)
–	Without this combination of characters	**11**
11	Aedeagal shaft moveably hinged basally or if not hinged (*Gurawa*) forewing without appendix; connective loop-shaped with arms closely appressed anteriorly; first valvula dorsal sculpturing maculate to granulose not reaching dorsal margin; second valvula with uniform-shaped teeth	**Chiasmini 12**
–	Without this combination of characters	**17**
12	Male pygofer with caudal marginal darkly sclerotised dentate crest	***Aconurella***
–	Pygofer not as above	**13**
13	Head spatulate, foremargin sharply angled in lateral view, carinate (Fig. 67)	**14**
–	Head not spatulate, foremargin rounded in lateral view (Fig. 68)	**15**
14	Forewing lacking appendix; ocelli near anterior margin of head (Fig. 67)	***Gurawa***
–	Forewing when fully developed with appendix (Fig. 59); ocelli on vertex some distant from anterior margin	***Chiasmus***
15	Opaque green (rarely blue) species with black markings	***Nephotettix***
–	Pale brown species with or without markings	**16**
16	Crown with or without transverse black band; male pygofer with few apical stout setae (Fig. 28)	***Exitianus***
–	Crown without transverse black band; male pygofer without apical stout setae (Fig. 27)	***Leofa***
17	Ocelli closer to eyes than laterofrontal sutures; body dorsoventrally flattened; aedeagus with pair of apical processes	**Hecalini 18**
–	Ocelli and laterofrontal sutures equidistant from eyes; body not dorsoventrally flattened; aedeagus with or without apical processes	**21**
18	Brown species; male pygofer with caudal marginal stout setae	***Glossocratus***
–	Pale to green species; male pygofer without caudal marginal stout setae	**19**
19	Crown with bold orange or yellow inverted V-shaped band, pronotum with two bold arcuate orange bands (Fig. 72); forewing with claval vein A1 merging with claval suture	***Linnavuoriellaarcuata*** (p. 160)
–	Crown without coloured bands or with bands subparallel or converging, but not very bold and not broadly contiguous at median line; pronotum with or without bands; forewing with A1 not merging with claval suture, but with two separate claval veins	**20**
20	Crown without orange or yellow colour pattern; tegmina unmarked (Fig. 8)	***Hecalus***
–	Crown with pair of orange or yellow longitudinal bands subparallel or converging, but not contiguous anteriorly, sometimes faint or absent; tegmina invariably with apical brown patch with white spots (Fig. 74)	***Thomsoniaporrecta***
21	Aedeagus with two shafts	**Opsiini 22**
–	Aedeagus with one shaft	**26**
22	Aedeagus with shafts fused in basal half of the length, apically divergent, forming a circle (Fig. 53)	**Neoaliturus (Circulifer)**
–	Aedeagal shaft fused basally but well separated throughout	**23**
23	Aedeagal shaft with apical or preapical processes (Fig. 44)	***Hishimonusphycitis*** (p. 165)
–	Aedeagal shaft without apical or preapical processes	**24**
24	Aedeagal shaft with pair of ventral processes	***Opsius***
–	Aedeagal shaft without pair of ventral processes	**25**
25	Crown, thorax and forewing with irregular brown maculation, pronotum and scutellum without red markings (Fig. 10)	***Orosius***
–	Crown sprinkled with fine dark brown spots, pronotum and scutellum with irregular red markings	.***Masiripiuslugubris*** (p. 165)
26	Connective fused to aedeagus	**Deltocephalini 27**
–	Connective articulated with aedeagus	**29**
27	Crown with transverse black stripe; male pygofer with appendage on dorsal margin	***Paramesodeslineaticollis*** (p. 156)
–	Crown without transverse black stripe; male pygofer without appendage on dorsal margin	**28**
28	Aedeagal shaft short, robust, strongly curved dorsally, with apical gonopore (Fig. 45)	***Deltocephalus***
–	Aedeagal shaft long, slightly curved dorsally, with gonopore indistinct (Fig. 46)	***Maiestas***
29	Forewings with two anteapical cells; preatrium of aedeagus without long processes (Fig. 60)	**Macrostelini 30**
–	Forewings with three anteapical cells, if with two anteapical cells then preatrium of aedeagus with two long processes	**32**
30	Head with crown of uniform length throughout width, more than four times broader than long (Fig. 12)	***Balclutha***
–	Crown distinctly longer medially than next to eyes, two times or less broader than median length	**31**
31	Pale yellow to brown or black in colour; male pygofer processes absent, caudal margin with comb-like serrations (Fig. 29)	***Macrosteles***
–	Golden yellow in colour, vertex with a pair of rounded dark brown spots; male pygofer with process present, caudal margin without comb-like serrations	***Cicadulina***
32	Male segment X elongate and sclerotised dorsally (Fig. 38)	**Cicadulini (*Pseudosubhimalus*)**
–	Male segment X not as above	**33**
33	Aedeagus with dorsal connective (Fig. 47)	**Limotettigini (Limotettix (Scleroracus) cacheolus)** (p. 161)
–	Aedeagus without dorsal connective	**34**
34	Connective with arms parallel (Fig. 54)	**Stenometopiini (*Stirellus*)**
–	Connective with arms not parallel	**35**
35	Frontoclypeus long and narrow (except *Monobazus*) (Fig. 65); male or female pygofer with dense tufts of either long fine or regular setae	**Scaphoideini 36**
–	Frontoclypeus broad (Fig. 66); male or female pygofer without dense tufts of long fine setae	**42**
36	Crown with distinct black spot near posterior margin (Fig. 75)	***Phlogotettixindicus***(p. 173)
–	Crown without distinct black spot near posterior margin	**37**
37	Brown species, forewing with whitish costal area (Fig. 15)	***Grammacephalus***
–	Brown to yellowish brown species, forewing without whitish costal area	**38**
38	Forewing with 3 or 4 crossveins extending to costal margin from outer apical cell (Fig. 61)	**39**
–	Forewing with at most 2 crossveins in costal region	**40**
39	Connective with paraphysis (Fig. 55); aedeagal shaft very short	***Scaphoideusharlani*** (p. 173)
–	Connective without paraphysis; aedeagal shaft elongate, cylindrical	***Bampuriuspakistanicus*** (p. 171)
40	Male subgenital pl. with mesal sclerotised process (Fig. 48)	***Neolimnusegyptiacus*** (p. 172)
–	Male subgenital pl. without mesal sclerotised process	**41**
41	Aedeagal shaft with processes arising on dorsal surface	***Monobazusdissimilis*** (p. 172)
–	Aedeagus with ventro-lateral processes	**Osbornellus (Mavromoustaca) macchiae** (p. 172)
42	Connective arms closely appressed anteriorly	**Paralimnini 43**
–	Connective arms not closely appressed anteriorly, divergent	**Athysanini (in part) 47**
43	Crown with pair of black anterior markings (Fig. 18)	***Changwhania***
–	Crown without pair of black markings	**44**
44	Anterior margin of crown with transverse black stripe (Fig. 19); connective V-shaped	***Paralimnuscingulatus***
–	Anterior margin of crown without transverse black stripe; connective Y-shaped	**45**
45	Subgenital plates short	***Psammotettixemarginatus***
–	Subgenital plates long	**46**
46	Anal tube with long process (Fig. 49); aedeagus with dorsal connective well-developed (Fig. 50)	***Jilinga***
–	Anal tube without process; aedeagus with dorsal connective absent	***Soractellusnigrominutus*** (p. 169)
47	Crown pointed anteriorly; aedeagus without apical lateral processes	***Platymetopius***
–	Crown rounded anteriorly; aedeagus with apical laterally directed small processes (Fig. 52)	***Euscelidiuscornix***

### ﻿Checklists and keys to species of Pakistani Deltocephalinae

Keys to all species of Pakistan Deltocephalinae are given for each genus containing more than one species. We follow Zahniser and Dietrich (2013) for most of the tribal diagnostic characters.

#### Athysanini Van Duzee

**Diagnosis.** It is impossible to provide a set of characters to easily diagnose this large tribe due to its morphological diversity. However, most members have the connective Y-shaped and lack the distinctive features of other tribes.

#### *Euscelidius* Ribaut

***E.cornix* Naveed & Zhang**

Figs 23, 36, 52

*Euscelidiuscornix* Naveed & Zhang, 2020c: 470, fig. 1A–G (Pakistan).

#### *Platymetopius* Burmeister

***Platymetopius* sp.**

**Remarks.** From the figure (code number DW 50A, unidentified) given by Mahmood (1969) this genus is present in Pakistan.

**Figures 1–15.**
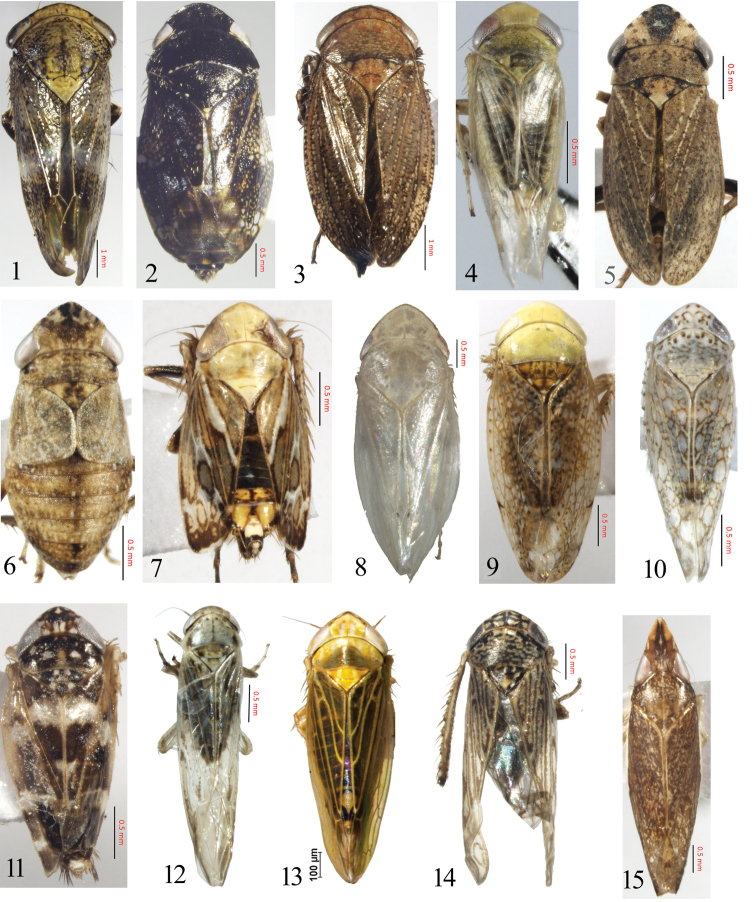
(habitus, dorsal view) **1***Drabescusangulatus***2***Neodartusacocephaloides***3**Goniagnathus (Tropicognathus) nepalicus**4***Aconurellaprolixa***5***Gurawaminorcephala***6***Chiasmus* sp. **7**Leofa (Prasutagus) pulchellus**8***Hecalusghaurii***9***Hishimonusphycitis***10***Orosiusaegypticus***11***Maiestasalbomaculata***12***Balcluthapunctata***13***Pseudosubhimaluspakistanicus***14**Limotettix (Scleroracus) cacheolus**15***Grammacephalusraunoi*.

**Figures 16–24.**
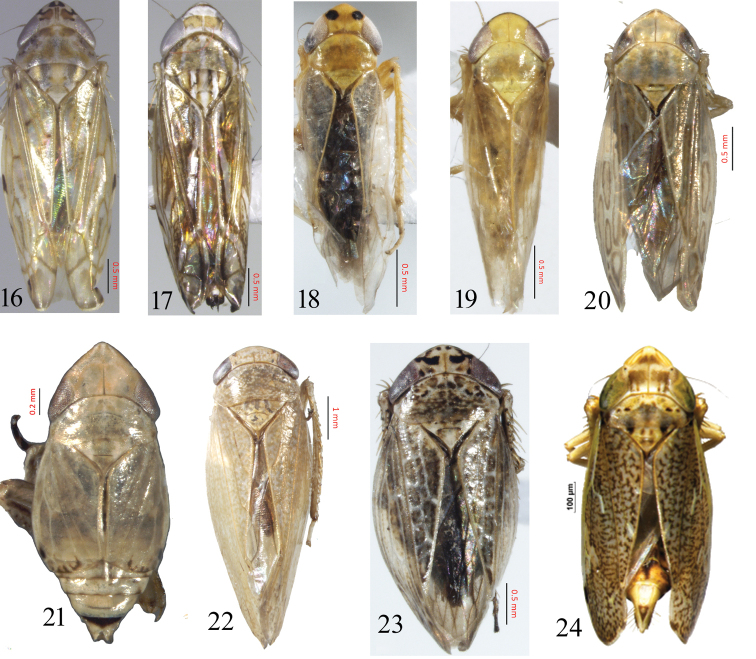
(habitus, dorsal view) **16***Neolimnusegyptiacus***17***Scaphoideusharlani***18***Changwhaniaterauchii***19***Paralimnelluscingulatus***20***Jilingatruncata***21***Soractellusnigrominutus***22***Tambocerusbulbous***23***Euscelidiuscornix***24***Stirellusmankiensis*.

#### *Tambocerus* Zhang & Webb

**Remarks.***Tambocerus* is one of the few Athysanini with transverse striations on the fore margin of the head.

***T.bulbulus* Naveed & Zhang**

Figs 22, 39, 51

*Tambocerusbulbulus* Naveed & Zhang, 2018i: 240, figs 3A–D, 4A–I (Pakistan).

#### Chiasmini Distant

**Diagnosis.** These are small to medium sized leafhoppers, usually white, stramineous, green, brown, grey, or black in colouration, and sometimes iridescent. They can be identified by the tapering or parallel sided clypellus, aedeagus hinged at the base (hinge usually but not always present), ovipositor usually extending far beyond the pygofer, first valvula dorsal sculpturing pattern maculate to granulose and usually submarginal, first valvula without distinctly delimited ventroapical sculpturing, and second valvula teeth obliquely triangular and serrated.

**Figures 40–55.**
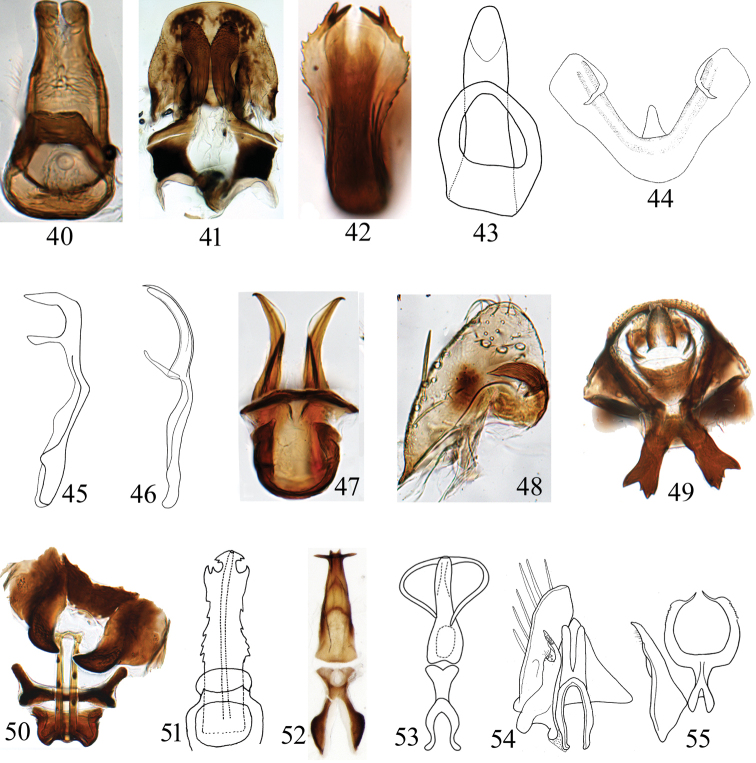
(male genitalia) **40***Neodartusacocephaloides* aedeagus, dorsal view **41**Goniagnathus (Tropicognathus) nepalicus fused subgenital plates and valve, styles and base of connective **42***Gurawaminorcephala* aedeagus, caudal view **43***Chiasmus* sp. aedeagus, dorsal view **44***Hishimonusphycitis* aedeagus, posterior view **45***Deltocephalusvulgaris* aedeagus and connective, lateral view **46***Maiestas* sp. aedeagus and connective, lateral view **47**Limotettix (Scleroracus) cacheolus aedeagus, dorsal view **48***Neolimnusegyptiacus* subgenital plate **49***Jilingatruncata* annal tube, ventral view **50***Jilingatruncata* aedeagus and dorsal connective, ventral view **51***Tambocerusbulbulus* aedeagus, posterior view **52***Euscelidiuscornix* aedeagus and connective, dorsal view **53**Neoaliturus (circulifer) tenellus aedeagus and connective **54***Stirelluslahorensis* valve, style, and connective, dorsal view **55***Scaphoideusharlani* connective and style.

**Figures 25–39.**
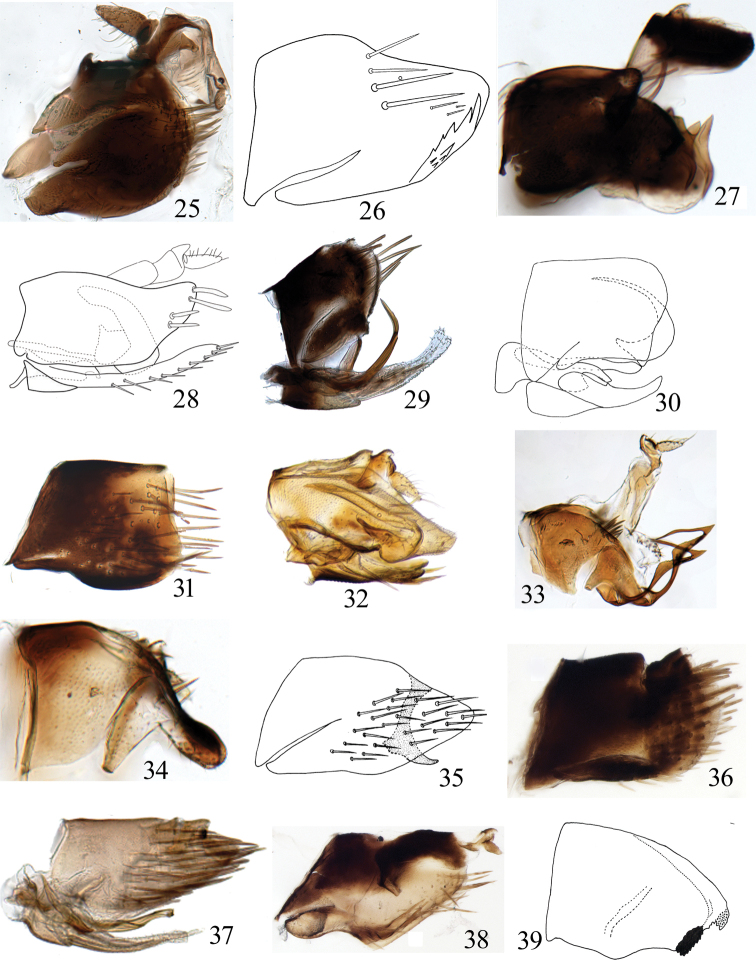
(male pygofer, lateral view) **25***Neodartusacocephaloides***26***Aconurellaprolixa***27**Leofa (Prasutagus) pulchellus**28***Exitianusnanus***29***Macrostelesparafalcatus***30***Balcluthapunctata***31***Jilingatruncata***32***Stirellusmankiensis***33***Grammacephalusraunoi***34***Neolimnusegyptiacus***35***Paralimnelluscingulatus***36***Euscelidiuscornix***37***Hecalusrawalakotensis***38***Pseudosubhimaluspakistanicus***39***Tambocerusbulbulus*.

**Figures 56–75.**
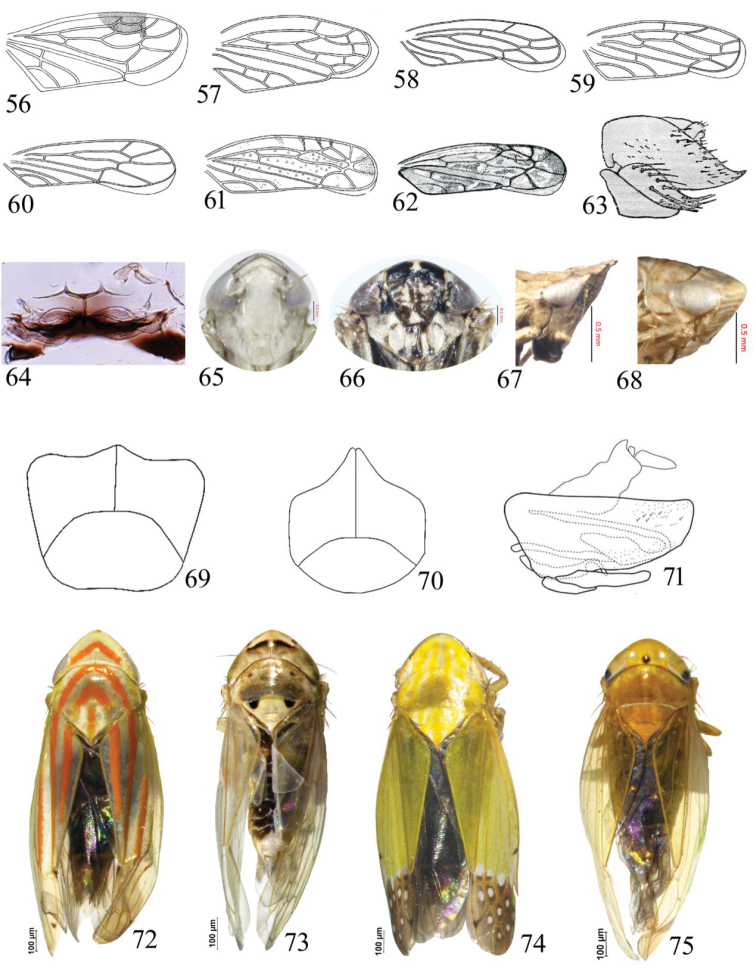
**56–62** (forewings) **56***Drabescusnitens***57**Goniagnathus (T.) quadripinnatus**58***Aconurellaprolixa***59***Chiasmus* sp. **60***Macrostelesindrinus***61***Bampuriuspakistanicus***62***Scaphoideusimmistus***63***Stirellusthattaensis*, pygofer, lateral view **64***Macrostelesparafalcatus*, male 2^nd^ abdominal tergites, dorsal view **65***Scaphoideusharlani*, face **66***Euscelidiuscornix*, face **67***Gurawalongispina*, head, lateral view **68***Leofanaga*, head, lateral view **69**Neoaliturus (C.) tenellus, subgenital plates **70**Neoaliturus (C.) opacipennis, subgenital plates **71***Stirellusviridulus*, pygofer, lateral view **72***Linnavuoriellaarcuata*, habitus, dorsal view **73***Exitianusnanus*, habitus, dorsal view **74***Thomsoniaporrecta*, habitus, dorsal view; **75***Phlogotettixindicus*, habitus, dorsal view.

#### *Aconurella* Ribaut

***A.choui* Naveed & Zhang**

*Aconurellachoui* Naveed & Zhang, 2018a: 72, fig. 5; pl. II, figs A–D (Pakistan).

***A.erebus* (Distant)**

*Deltocephaluserebus* Distant, 1908: 385 (India).

*Aconurellaerebus*: Ghauri, 1974: 553–555, figs 14–17 (India).

*Aconurellaerebus*: Naveed and Zhang 2018a: 68, fig. 2; pl. I, figs D–F (Pakistan).

***A.naranensis* Naveed & Zhang**

*Aconurellanaranensis* Naveed & Zhang, 2018a: 71, fig. 4; pl. I, J–L (Pakistan).

***A.paraerebus* Naveed & Zhang**

*Aconurellaparaerebus* Naveed & Zhang, 2018a: 68, fig. 3; pl. I, G–I (Pakistan).

***A.prolixa* (Lethierry)**

Figs 4, 26, 58

*Thamnotettixprolixa* Lethierry, 1885: 102 (Europe).

*Thamnotettixminutes* Haupt, 1917: 254. Synonymised by Dlabola 1963: 324.

*Thamnotettixsanguisuga* Lindberg, 1927: 88. Synonymised by Metcalf 1967a: 1597.

*Cicadulaindica* Pruthi, 1930: 54. Synonymised by Khatri and Webb 2010: 9 (India).

*Deltocephalusobtusus* Metcalf, 1955: 266. (nom. nov. for *Deltocephalussimplex* Haupt, 1927, non *D.simplex* Van Duzee, 1892: 304).

*Chiasmuskarachiensis*Ahmed et al., 1988: 13, fig. 3A–J. Synonymised by Khatri and Webb 2010: 9 (Pakistan).

*Chiasmuslobata*Ahmed et al., 1988: 14, fig. 4A–J. Synonymised by Khatri and Webb 2010: 9.

*Aconurellaneosolana* Rao & Ramakrishnan, 1990a: 268, fig. 1 (India). Synonymised by Khatri and Webb 2010: 9.

*Aconurellaprolixa* Khatri & Webb, 2010: 4, pl. 1, fig. g; fig. 9; Naveed and Zhang 2018a: 67, fig. 1; pl. I, A–C (Pakistan).

### ﻿Key to *Aconurella* species (male) modified from Naveed and Zhang (2018a)

**Table:** 

1	Pygofer side with many spinules at dorsoapical margin, some large	**2**
–	Pygofer side dorsoapical margin without or with sparse small spinules	**4**
2	Subgenital plates as long as pygofer; with two macrosetae at apex	***A.paraerebus***
–	Subgenital plates subequal to pygofer; with more than two macrosetae at apex	**3**
3	Subgenital plates longer than pygofer; style apophysis smooth	***A.erebus***
–	Subgenital plates shorter than pygofer; style apophysis serrate with enlarged preapical tooth	***A.naranensis***
4	Pygofer dorsal margin without spinules (Fig. 26); connective arms close together distally	***A.prolixa***
–	Pygofer dorsal margin with small spinules; connective arms widely separate from each other	***A.choui***

#### *Chiasmus* Mulsant & Rey

***C.alatus* Pruthi**

*Chiasmusalatus* Pruthi, 1930: 23, pl. II, figs 6, 6a, text figs 32–34 (India); Khatri and Webb 2010: 4 (Pakistan).

***C.niger* Pruthi**

*Chiasmusniger* Pruthi, 1936: 108, pl. VIII, fig. 8, text fig. 122 (India); Khatri and Webb 2010: 4 (Pakistan).

**Remarks.** The identification key of this species has not been possible due to the uncertainty of the differences between very similar species. The previously described forms may prove to be synonyms.

#### *Exitianus* Ball

***E.indicus* (Distant)**

*Athysanusindicus* Distant, 1908: 344 (India).

*Athysanusatkinsoni* Distant, 1908: 345 (India). Synonymised by Ross, 1968: 12.

*Exitianusindicus*: Ross 1968: 12, figs 9, 10, 26–30, 69.

*Exitianusmajor*Ahmed et al., 1988: 10, fig. 1 (Pakistan). Synonymised by Khatri and Webb 2010: 10.

*Exitianusindicus*: Duan and Zhang 2013: 36, pl. II, figs 3–6; Khatri et al. 2014: 3, pl. 1 (China).

***E.nanus* (Distant)**

Fig. 73

*Athysanusnanus* Distant, 1908: 345 (India).

*Athysanusinsularis* Distant, 1909: 47, pl. 4, figs 10, 10a. Synonymised by Ross 1968: 7.

*Athysanusfasciolatus* Melichar, 1911: 107 (East Africa). Synonymised by Linnavuori 1975: 626.

*Athysanussimillimus* Matsumura, 1914: 185 (Japan). Synonymised by Ross 1968: 7.

*Athysanusvulnerans* Bergevin, 1925: 42, figs 5–9 (East Africa). Synonymised by Ross 1968: 7.

*Limotettixalbipennis* Haupt, 1927: 25, pl. II, figs 20a–c (Palestine). Synonymised by Dlabola 1963: 325.

*Limotettixunifasciata* Haupt, 1930: 159, fig. 9. Synonymised by Dlabola 1963: 325.

*Athysanusdigressus* Van Duzee, 1933: 32 (USA). Synonymised by Linnavuoriand DeLong 1978: 237.

*Exitianusnanus*: Ross, 1968: 7, figs 1–3, 15–18, 76; Duan and Zhang 2013: 33, pl.pl. I, figs 1–2 (China); Khatri et al. 2014: 4; Duan and Zhang 2013: 33, pl. I, figs 1, 2; Khatri et al. 2014: 3, pl. 2 (Pakistan).

*Exitianuskarachiensis* Ahmed, 1986: 59, fig. 5. Synonymised by Khatri and Webb 2010: 10.

*Exitianuspeshawarensis* Ahmed & Rao, 1986: 76–77, fig. 1. Synonymised by Khatri and Webb 2010: 10.

*Exitianusminor*Ahmed et al., 1988: 12, fig. 2. Synonymised by Khatri and Webb 2010: 10.

*Exitianusfulvinervis* Li & He, 1993: 27; Li et al. 2011: 68, fig. 55. Synonymised by Duan and Zhang 2013: 33 (China).

### ﻿Key to *Exitianus* species from Pakistan (male)

**Table:** 

1	Crown with transverse brown band usually interrupted medially (Fig. 73); pygofer side with 2–6 apical brown or black macrosetae	***E.nanus***
–	Crown with transverse brown band usually complete; pygofer side with 2 or 3 apical brown or black macrosetae	***E.indicus***

#### *Gurawa* Distant

***G.minorcephala* Pruthi**

Fig. 5

*Gurawaminorcephala* Pruthi, 1930: 29, pl. II, fig. 10a, b, text figs 41,42 (Pakistan); Zahniser 2008: 22, figs 77–85; Dai et al. 2011: 38, fig. 1; Duan and Zhang 2012: 42–44, pl. I, fig. 1 (China); Viraktamath and Gnaneswaran 2013: 199–200, figs 22–29, 41, 55–58 (India); Naveed and Zhang 2018b: 482, figs 1E–H, 2A–G, 4A–E, 5B (Pakistan).

***G.longispina* Naveed & Zhang**

*Gurawalongispina* Naveed & Zhang, 2018b: 486, figs 1A–D, 3A–F, 5A (Pakistan).

### ﻿Key to *Gurawa* species from Pakistan (male) modified from Naveed and Zhang 2018b

**Table:** 

1	Crown with dorsal constriction at level of ocelli; aedeagal shaft with lateroapical spines long in posterodorsal view	***G.longispina***
–	Crown without dorsal constriction at level of ocelli; aedeagal shaft with lateroapical spines short in posterodorsal view	***G.minorcephala***

#### *Leofa* Distant

### ﻿Key to subgenera of *Leofa* from Pakistan modified from Naveed and Zhang (2018c)

**Table:** 

1	Submacropterous; pygofer with a well-developed dorsal appendage	**Leofa (Prasutagus)**
–	Brachypterous; pygofer without dorsal appendage	**Leofa (Leofa)**

**L. (L.) mysorensis Distant**

*Leofamysorensis* Distant, 1918: 86; Viraktamath and Viraktamath 1992: 5, figs 10–19 (India); Naveed and Zhang 2018c: 46, figs 5–8 (Pakistan).

*Leofaaffinis* Distant, 1918: 87. Synonymised by Viraktamath and Viraktamath 1992: 5 (India).

*Leofasanguinalis* Distant, 1918: 87. Synonymised by Viraktamath and Viraktamath 1992: 5 (India).

*Leofaunicolor* Distant, 1918: 88. Synonymised by Viraktamath and Viraktamath 1992: 5 (India).

*Leofapedestris* Distant, 1918: 88. Synonymised by Viraktamath and Viraktamath 1992: 5 (India).

*Leofaparwala* Pruthi, 1930: 26. Synonymised by Viraktamath and Viraktamath 1992: 5 (India).

**L. (L.) naga Viraktamath & Viraktamath**

*Leofanaga* Viraktamath & Viraktamath, 1992: 9–10, figs 31–40 (India); Naveed and Zhang 2018c: 46, figs 9–13 (Pakistan).

**L. (Prasutagus) pulchellus Distant**

Figs 7, 27

*Prasutaguspulchellus* Distant, 1918: 53–54, fig. 57 (India).

Leofa (Prasutagus) pulchellus: Zahniser, 2008: 18; Duan et al. 2012: 39 (China); Naveed and Zhang 2018c: 46, figs 1–4 (Pakistan).

**L. (L.) truncata Viraktamath & Viraktamath**

*Leofatruncata* Viraktamath & Viraktamath, 1992: 4, figs 1–9 (India); Naveed and Zhang 2018c: 47, 14–19 (Pakistan).

### ﻿Key to *Leofa* species from Pakistan (male)

**Table:** 

1	Subgenital plates rounded caudally; pygofer with or without shallow lateral furrow; aedeagal shaft with caudal hood, basal process short, narrower than width of shaft	**2**
–	Subgenital plates truncate caudally; pygofer deeply furrowed laterally; aedeagal shaft without caudal hood, basal process long, broader than width of shaft	***L.truncata***
2	Aedeagal shaft tubular, without lamellate expansion; gonopore slightly asymmetrically placed on left side; caudal hood not strongly developed	***L.mysorensis***
–	Aedeagal shaft hood-like with lateral lamellate expansion; caudal hood strongly developed; gonopore symmetrically placed	***L.naga***

#### *Nephotettix* Matsumura

***N.nigropictus* (Stål)**

*Thamnotettixnigropictus* Stål, 1870: 740 (India).

*Nephotettixapicalis* Distant, 1908: 360 (India); Ishihara 1964: 42; Ishihara and Kawase 1968: 123.

*Nephotettixnigropictusyapicola* Ghauri, 1971: 495.

*Nephotettixnigropictus*: Ghauri, 1971: 491; Vilbaste 1975: 233; Ramakrishnan and Ghauri 1979; Mahmood and Aziz 1979: 61, figs 1b, 3a–f (Pakistan); Duan and Zhang 2014: 219, pl. III; pl. VI: I–L; figs 14, 15 (China).

***N.parvus* Ishihara & Kawase**

*Nephotettixparvus* Ishihara & Kawase, 1968: 121 (Japan); Duan and Zhang 2014: 221, pl. IV, pl.VIIA–C; fig. 16 (China).

*Nephotettixolivacea* Mahmood & Aziz, 1979: 65 (Pakistan). Synonymised by Wilson 1989: 136.

***N.virescens* (Distant)**

*Selenocephalusvirescens* Distant, 1908: 291 (India).

*Phrynomorphusolivacescens* Distant, 1918: 52. Synonymized by Wilson 1989: 135.

*Nephotettixbipunctatus* (Fabricius), Distant, 1908: 359.

*Nephotettiximpicticeps* Ishihara, 1964: 42. Synonymized by Ghauri,1971: 484.

*Nephotettixvirescens*: Ghauri, 1971: 484; Ramakrishnan and Ghauri 1979: 357; Duan and Zhang 2014: 223, pl. V; pl. VII: D–F; figs 17–18 (China).

*Nephotettixoryzii* Mahmood & Aziz, 1979: 63 (Pakistan). Synonymized by Wilson 1989: 135.

### ﻿Key to species of *Nephotettix* (male)

**Table:** 

1	Crown without traces of marginal and submarginal black transverse bands in both sexes	***N.virescens***
–	Crown with black submarginal transverse band markedly and fully developed	**2**
2	Anterior margin of pronotum marked with black transverse band	***N.nigropictus***
–	Anterior margin of pronotum without black markings	***N.parvus***

#### Cicadulini Van Duzee

**Diagnosis.**Cicadulini, following Zahniser and Dietrich (2013: 56), is a rather poorly defined tribe. It was defined by these authors in the following way: “small to medium sized, slender, stramineous, yellow, or greenish leafhoppers, sometimes with the anterior margin of the head marked with black spots. They can be identified by the male segment X often long and strongly sclerotised, and subgenital plates sometimes with a row of macrosetae near the middle and with long fine setae laterally” and additionally in their key: “male pygofer incised dorsally nearly to base”. Clearly, this definition is not ideal as you may not be able to identify a taxon (for example in a key) based solely on “often” and “sometimes” characters and also in their figure 15 of *Cicadula* Zetterstedt, segment X is moderately long (although the dorsal pygofer incision is very long and therefore the dorsal bridge very short). In addition, the genus *Pseudosubhimalus* Ghauri, placed in Athysanini by Zahniser and Dietrich (2014), was subsequently placed in Cicadulini based on molecular evidence and (in its type species) segment X is long and well sclerotised (Meshram and Niranjana 2019) However, in the genus the subgenital plate macrosetae are marginal, and in one of its species, *P.katraini* Meshram and Niranjana, segment X is very short. Similarly, segment X is not elongate in the Nearctic *Knullana* DeLong. The following three species of this genus occur in Pakistan.

#### *Pseudosubhimalus* Ghauri

***P.bicolor* (Pruthi)**

*Ophiolabicolor* Pruthi, 1936: 123 (India).

*Pseudosubhimalusbicolor*: Ghauri, 1974: 553; Meshram and Niranjana 2019: 7–9, figs 1A, 1B, 1E, 1G–1L, 2A–2F, 3A–3H (India, Pakistan).

***P.trilobatus* Meshram & Niranjana**

*Pseudosubhimalustrilobatus* Meshram & Niranjana, 2019: 7, 11–12, figs 1C, 1D, 4A–4F (India).

*Pseudosubhimalusbicolor* (Pruthi): Menghwar et al. 2015: 142, pl. 1, figs a-h (misidentification) (Pakistan).

***P.pakistanicus* Naveed & Zhang**

Figs 13, 38

*Pseudosubhimaluspakistanicus*Naveed et al., 2020a: 194, fig. 1A–H (Pakistan).

### ﻿Key to *Pseudosubhimalus* species from Pakistan (male) modified from Naveed et al. (2020a)

**Table:** 

1	Greyish green to pale yellow species, disc of crown without black or dark brown spots; pygofer lobe with weak ventral process (Fig. 38)	***P.pakistanicus***
–	Dark brown in colour, disc of crown with black or dark brown spots; pygofer lobe without ventral process	**2**
2	Pygofer ventral margin with dentations	***P.bicolor***
–	Pygofer ventral margin without dentations, smooth	***P.trilobatus***

#### Deltocephalini Fieber

**Diagnosis.** The members of this tribe are small to medium sized leafhoppers and are variable in colour. They can be identified by the tapering or parallel-sided clypellus, narrow lorum, linear connective with anterior arms closely appressed, connective fused to the aedeagus, and first valvula dorsal sculpturing imbricate (Scale-like).

#### *Deltocephalus* Burmeister

***D.vulgaris* Dash & Viraktamath**

Fig. 45

Deltocephalus (Deltocephalus) vulgaris Dash & Viraktamath, 1998: 4, figs 1–11 (India); Zhang and Duan 2011: 3, fig. 3A–H (China); Deltocephalus (Deltocephalus) vulgaris: Naveed et al. 2019a: 285, figs 1A, B, 3A–D (Pakistan).

***D.infirmus* Melichar**

*Deltocephalusinfirmus* Melichar, 1903: 203, pl. V, fig. 11 (Sri Lanka).

*Jassargusinfirmus*: Ishihara, 1961: 244, figs 53–58 (misidentification).

*Deltocephalusinfirmus*: Webb and Viraktamath 2009: 13, fig. 10; Naveed et al. 2019a: 285, figs 1C, 3D–G (Pakistan).

### ﻿Key to *Deltocephalus* species from Pakistan (male) modified from Naveed et al. (2019a)

**Table:** 

1	Crown with six brown spots on anterior margin; aedeagal shaft with shallow apical notch	***D.vulgaris***
–	Crown with single brown spot on anterior margin adjacent to eyes; aedeagal shaft without apical notch	***D.infirmus***

#### *Maiestas* Distant

***M.albomaculata* (Dash & Viraktamath)**

Fig. 11

Deltocephalus (Recilia) albomaculatus: Dash and Viraktamath 1998: 12, figs 29–34 (India).

*Maiestasalbomaculata*: Webb and Viraktamath 2009; Naveed et al. 2019a: 287, figs 1E–1I, 3H–3I; Shah et al. 2021: 403, figs 1A–D (Pakistan).

***M.indica* (Pruthi)**

*Allophlepsindica* Pruthi, 1936: 120–121, pl. IX, fig. 3, text fig. 132 (Pakistan); Rao and Ramakrishnan 1990: 111 (India).

Deltocephalus (Recilia) indicus: Dash and Viraktamath 1998: 35–36, fig. 305 (India).

*Maiestasindica*: Webb and Viraktamath 2009: 22; Shah et al. 2021: 403, fig. 1E (Pakistan).

***M.maculata* (Pruthi)**

*Cicadulamaculata* Pruthi, 1930: 58–59, figs 80–81, pl. V, fig. 2 (India).

*Thamnotettixprabha* Pruthi, 1930: 62, figs 85, 86, pl. V, figs 6, 6a (India). Synonymized by Webb and Viraktamath 2009: 41.

*Reciliaprabha*: Ghauri, 1980: 166–169, figs 1, 3–11.

Deltocephalus (Recilia) maculata: Dash and Viraktamath 1998: 32, figs 260–269 (India).

*Maiestasmaculata*: Webb and Viraktamath 2009: 22, comb. nov.; Zhang and Duan 2011: 37–39, figs 33–35, pl. IV: E, pl. V: P, pl. VI: P (China); Shah et al. 2021: 404, fig. 2A–I (Pakistan).

***M.pruthii* (Metcalf)**

*Deltocephalusnotatus* Pruthi, 1936: 128–129, text fig. 139, pl. IX, fig. 10 (Pakistan). Preoccupied, not Melichar 1896.

*Deltocephaluspruthii* (Metcalf, 1967b: 1173, new name).

*Maiestaspruthii*: Webb and Viraktamath 2009: 20; Naveed et al. 2019a: 288, figs 2A–2C, 3J–3K; Shah et al. 2021: 4F–L (Pakistan).

***M.setosa* (Ahmed, Murtaza & Malik)**

*Reciliasetose*Ahmed et al., 1988: 412–414, fig. 2 (Pakistan).

*Maiestassetosa*: Webb and Viraktamath 2009: 20 (Pakistan).

***Maiestassinuata* Shah & Duan**

*Maiestassinuata* Shah & Duan, 2021: 406, fig. 3A–H (Pakistan).

***M.subviridis* (Metcalf)**

*Stirellussubviridis* Metcalf, 1946: 125. Synonymized with *S.hopponis* (Matsumura) by Linnavuori, 1975: 617, in error;

Deltocephalus (Recilia) subviridis: Dash and Viraktamath 1998: 24, figs 166–172 (India);

*Maiestassubviridis*: Webb and Viraktamath 2009: 19, fig. 40; Khatri and Webb 2010: 11, pl. 2b, c, fig. 12 (Pakistan); Zhang and Duan 2011: 19 (China); Shah et al. 2021: 408, fig. 4A–E (Pakistan).

***M.tareni* (Dash & Viraktamath)**

Deltocephalus (Recilia) tareni Dash & Viraktamath, 1995: 74–76, figs 1–15; Dash and Viraktamath 1998: 16, figs 78–84 (India).

*Maiestastareni*: Webb & Viraktamath, 2009: 22; Khatri and Webb 2010: 11, pl. 2d, fig. 11 (Pakistan); Zhang and Duan 2011: 20 (China); Naveed et al. 2019a: 288, figs 2G–I, 3N–3O; Shah et al. 2021: 408, fig. 5A–H (Pakistan).

***Maiestastrispinosa* (Dash & Viraktamath)**

Deltocephalus (Recilia) trispinosus Dash & Viraktamath, 1998: 35, figs 296–304 (India).

*Maiestastrispinosa*: Webb and Viraktamath 2009: 38; Shah et al. 2021: 408, fig. 6A–I (Pakistan).

### ﻿Key to *Maiestas* species from Pakistan (male). *Maiestassetosa* is excluded from the key due to the poor original description and figures.

**Table:** 

1	Overall colour dark brown; forewing with sub-basal and subapical irregular white transverse band (Fig. 11)	***M.albomaculata***
–	Colour not as above	**2**
2	Crown, face and thorax with black patches	***M.maculata***
–	Crown, face and thorax without black patches	**3**
3	Forewing with extra cross-veins, at least in clavus	**4**
–	Forewing without extra cross-veins	**5**
4	Aedeagus with a large subapical ventral process	***M.indica***
–	Aedeagus with a short apical ventral process	***M.pruthii***
5	Aedeagus with pair of short lateral processes	***M.trispinosa***
–	Aedeagus without lateral processes	**6**
6	Aedeagus in lateral view similar in width in distal half	***M.subviridis***
–	Aedeagus in lateral view evenly tapered from base to apex	**7**
7	Style apophysis broadest sub-basally; aedeagal shaft in lateral view not sinuate	***M.tareni***
–	Style apophysis broadest at base; aedeagal shaft in lateral view slightly sinuate	***M.sinuata***

#### *Paramesodes* Ishihara

***P.lineaticollis* (Distant)**

*Paramesodeslineaticollis* (Distant, 1908: 294, *Paramesus*) (India); Wilson 1983: 21–22, figs 23–29.

*Paramesodesishurdii* Mahmood & Meher, 1973: 135 (Pakistan). Synonymised by Wilson 1983: 21.

#### Drabescini Ishihara

**Diagnosis.**Drabescini are medium sized to large leafhoppers, variable in colour and shape. They can be identified by the following combination of characters: antennae long situated near upper part of face; antennal pits large, often encroaching onto frontoclypeus; anterior margin of head smooth, irregularly textured, or with one to many carinae or striae; nymph often with apical process on head. Two subtribes are present (see key and below).

#### Drabescina

***Drabescus* Stål**

***D.angulatus* Signoret**

Fig. 1

*Drabescusangulatus* Signoret, 1880: 210; Ghauri 1965: 688; Zhang and Webb 1996: 24, figs 380–384, 525.

#### Paraboloponina Ishihara

***Dryadomorpha* Kirkaldy**

**Remarks.** See Zhang and Webb (1996: 6) for full synonymy.

***D.pallida* Kirkaldy**

*D.pallida* Kirkaldy, 1906: 336; Webb 1981: 50–53, figs 41–56.

**Remarks.** See Zhang and Webb (1996: 14) for full synonymy.

#### Goniagnathini Wagner

**Diagnosis.** These are medium sized to large, squat, robust leafhoppers. They can be identified by the short and broad head, anterior margin of head glabrous, large forewing appendix (in macropterous individuals), subgenital plates fused to each other, valve apparently absent or fused to subgenital plates, style with broad basal part articulated with linear or modified apical part, and connective fused to the aedeagus.

#### *Goniagnathus* Fieber

**G. (Epistagma) guttulinervis (Kirschbaum)**

Jassus (Athysanus) guttulinervis Kirschbaum, 1868: 116 (Europe).

*Thamnotettixputoni* Lethierry, 1874: 444.

*Goniagnathusocellatus* Jacobi, 1910: 133.

*Goniagnathusguttulinervis*: Dash and Viraktamath 2001: 64, figs 1–5 (India); Naveed and Zhang 2018j: 1805, fig. 1C; Shah and Duan 2020b: 16–17, figs 1A, B, 2A–H (Pakistan).

**G. (Tropicognathus) nepalicus Viraktamath & Gnaneswaran**

Fig. 3

Goniagnathus (Tropicognathus) nepalicus Viraktamath & Gnaneswaran, 2009: 56–57, figs 5, 6, 19–24 (Nepal); Naveed and Zhang 2018j: 1806, figs 1E–G; Shah and Duan 2020b: 16, 20, figs 1E, 1F, 5A–D (Pakistan).

**G. (Tropicognathus) punctifer (Walker)**

*Bythoscopuspunctifer* Walker, 1858: 104.

*Goniagnathuselongatus* Lethierry, 1892: 209.

*Goniagnathusspurcatus*: Melichar 1903: 181.

*Goniagnathuspunctifer*: Distant 1908: 311; Zhang 1990: 91; Dash and Viraktamath 2001: 71 (India).

Goniagnathus (Tropicognathus) punctifer: Duan and Zhang 2009: 53, figs 2A–E, 7E, 7K, 8D (China); Shah and Duan 2020b: 19, figs 6–8 (Pakistan).

**G. (Tropicognathus) quadripinnatus Dash & Viraktamath**

Goniagnathus (Tropicognathus) quadripinnatus Dash & Viraktamath, 2001: 74–76, figs 45–50 (India); Naveed and Zhang 2018j: 1806, fig. 1D; Shah et al. 2020b: 16, figs 1C, 1D, 3A–G (Pakistan).

### ﻿Key to subgenera and species of *Goniagnathus* from Pakistan (male) modified from Shah et al. (2020)

**Table:** 

1	Male pygofer with dorsal appendage absent; aedeagus with pair of ventral processes exceeding aedeagal shaft	**G. (Epistagma) guttulinervis**
–	Male pygofer with dorsal appendage present; aedeagus with pair of ventral processes not exceeding aedeagal shaft	**G. (Tropicognathus) 2**
2	Aedeagus with one pair of long processes present at mid-length, subgenital plates fused with truncate margin caudally	**G. (Tropicognathus) nepalicus**
–	Aedeagus with two pairs of processes	**3**
3	Aedeagal shaft with a pair of apical and a pair of median asymmetrical processes	**G. (Tropicognathus) punctifer**
–	Aedeagal shaft with two pairs of processes present near apex, having lateral processes longer and stouter than the dorsal processes	**G. (Tropicognathus) quadripinnatus**

#### Hecalini Distant

**Remarks.** A revision of Oriental Hecalini was given by Morrison (1973).

**Diagnosis**. The members of this tribe are medium sized to large, somewhat to strongly dorsoventrally flattened, stramineous, yellow, green, or brown leafhoppers, sometimes with bright orange or reddish markings. They can be identified by the produced and parabolically shaped head, dorsoventrally flattened body, lateral margin of pronotum as long as or longer than the basal width of eye, ocelli closer to eyes than laterofrontal sutures, apodemes of male sternite I long and relatively narrow, apodemes of male sternite II broad and well-developed, male pygofer often produced or pointed posterodorsally, segment X withdrawn into pygofer, ventral margins of male pygofer often lobate, aedeagus often with one or two pairs of apical processes, first valvula dorsal sculpturing granulose to maculate and submarginal, first valvula often with distinctly delimited ventroapical sculpturing, second valvula usually without teeth, humpbacked dorsally, and concave ventrally.

#### *Glossocratus* Fieber

***Glossocratus* sp.**

**Remarks.** From the figure (unidentified) given by Mahmood (1979) this genus is present in Pakistan. No information is given by Mahmood on examined specimens.

#### *Hecalus* Stål

***H.erectus* Naveed & Zhang**

*Hecaluserectus* Naveed & Zhang, 2018d: 581, fig. 1A–H; pl. IA–C (Pakistan).

***H.ghaurii* Rao & Ramakrishnan**

Fig. 8

*Hecalusghaurii* Rao & Ramakrishnan, 1990b: 388, figs 1–11 (India); Naveed and Zhang 2018d: 584, fig. 2A–K; pl. ID–G (Pakistan).

***H.muzaffarabadensis* Naveed & Zhang**

*Hecalusmuzaffarabadensis* Naveed & Zhang, 2018d: 585, fig. 3A–D; pl. I, figs H–J (Pakistan).

***H.prasinus* (Matsumura)**

*Parabolocratusprasinus* Matsumura, 1905: 48 (Japan); Morrison 1973: 417, figs 154–159 (Thailand); Mahmood 1979: 93 (Pakistan).

***H.rawalakotensis* Naveed & Zhang**

*Hecalusrawalakotensis* Naveed & Zhang, 2019c: 596, figs 1A–I, 2A–D (Pakistan).

***H.snipus* Naveed and Zhang**

*Hecalussnipus* Naveed & Zhang, 2018d: 386, fig. 4A–G; pl. II, figs A–C (Pakistan).

***H.umballaensis* Distant**

*Hecalusumballaensis* Distant, 1908: 274; Morrison 1973: 431, fig. 190; Rao and Ramakrishnan 1990b: 390, figs 31–38 (India); Naveed and Zhang 2018d: 587, fig. 5A–I; pl. II, figs D–F (Pakistan).

***H.veracious* Naveed & Zhang**

*Hecalusveracious* Naveed & Zhang, 2018d: 587, fig. 6A–H; pl. II, figs G–I (Pakistan).

### ﻿Key to *Hecalus* species from Pakistan (male) modified from Naveed and Zhang (2018d) and Naveed et al. (2019c)

**Table:** 

1	Greenish brown to dark in colouration on face and thorax	**2**
–	Yellowish green to pale yellow in colouration on face and thorax	**3**
2	Aedeagal shaft with long, leaf-like, pointed apical processes	***H.umballaensis***
–	Aedeagal shaft with short, truncate apical processes	***H.snipus***
3	Aedeagal shaft with subapical dorsal flares and bifurcated apical processes	***H.muzaffarabadensis***
–	Aedeagal shaft without apical bifurcated processes	**4**
4	Aedeagal shaft without lateral serrations	***H.ghaurii***
–	Aedeagal shaft with lateral serrations	**5**
5	Aedeagal shaft with lateral serrations throughout	***H.erectus***
–	Aedeagal shaft with lateral serrations limited to basal 2/3	***6***
6	Aedeagal shaft nearly parallel sided throughout length in dorsal view	***H.veracious***
–	Aedeagal shaft broad in basal half, narrowed apically in dorsal view	***H.rawalakotensis***

#### *Linnavuoriella* Evans

***L.arcuata* (Motschulsky)**

Fig. 72

*Platymetopiusarcuatus*: Motschulsky, 1859: 115.

*Tetigoniakalidasa* Kirkaldy, 1900: 294.

*Parabolocratuscitrinus* Evans, 1941: 36.

*Vartamoshiensis* Rao, 1973: 96 (India).

*Hecalusarcuatus*: Morrison 1973: 426.

*Linnavuoriellaarcuata*: Hamilton 2000: 454; Catanach and Dietrich 2017; Naveed and Zhang 2019b: 619, fig. 2A–H (Pakistan); He et al. 2019: 267, figs 52–68 (China).

#### *Thomsonia* Signoret

***T.porrecta* (Walker)**

Fig. 74

*Acocephalusporrectus* Walker, 1858: 362.

*Platymetopiuslineolatus* Motschulsky, 1859: 114.

*Hecaluskirschbaumii* Stål, 1870: 737.

*Thomsoniellaalbomaculata* Distant, 1908: 278, fig. 178.

*Parabolocratusmerino* Capco, 1959: 333.

*Thomsoniellaporrecta*: Hamilton 2000: 454.

*Thomsoniaporrecta*: He et al. 2019: 269, figs 69–85 (China).

#### Koebeliini Baker

**Diagnosis.** These are small to medium sized, yellow, light green or brown leafhoppers. They can be identified by the combination of following characters: ocelli distant from eyes, clypellus long, narrow and extending well beyond normal curve of gena, and metatarsomere I with platellae on plantar surface.

#### *Pinopona* Viraktamath & Sohi

***P.minuta* Viraktamath & Sohi**

*Pinoponaminuta* Viraktamath & Sohi, 1998: 114, figs 1–15 (India, Nepal).

#### *Sohipona* Ghauri & Viraktamath

***S.webbi* Ghauri & Viraktamath**

*Sohiponawebbi* Ghauri & Viraktamath, 1987: 50, figs 11–29 (Pakistan).

#### Limotettigini Baker

**Diagnosis.** These are small to medium sized ivory, greyish, or black leafhoppers, often with dark markings. They can be identified by the parallel-sided or tapering clypellus, pygofer dorsal margin with spine-like process and aedeagus articulated with plate-like “dorsal connective” at dorsal margin of socle.

#### *Limotettix* Sahlberg

**Limotettix (Scleroracus) Van Duzee**

**L. (S.) cacheolus (Ball)**

Fig. 14

*Ophiolastratula* var. *cacheola* Ball, 1928: 189.

Limotettix (Scleroracus) cacheolus: Oman 1947: 205; Hamilton 1994: 122; McKamey 2001: 705 (USA); Naveed and Zhang 2018f: 79, figs 15–26 (Pakistan).

#### Macrostelini Kirkaldy

**Diagnosis.**Macrostelini are small to medium sized, slender, often stramineous, yellow, or greenish leafhoppers, with or without dark markings. They can be identified by their long, slender shape, forewing with two anteapical cells, subgenital plates usually with membranous digitate apical lobe, and male pygofer macrosetae sometimes plumose.

#### *Balclutha* Kirkaldy

***B.incisa* (Matsumura)**

*Gnathodusincisa* Matsumura, 1902: 360 (Japan).

*Balcluthaindica* Pruthi, 1930: 48, pl. IV, figs 4, 4a, 4b, text figs 67, 68 (*Eugnathodus*), India. Synonymised by Knight 1987: 1206.

*Balcluthaincisa*: Knight 1987: 1206, figs 138–145; Webb and Vilbaste 1994: 72, figs 10–17; Chiang 1996: 67, fig. 3; Dai, Li and Chen 2004: 749 (China); Naveed and Zhang 2018e: 259, fig. 2A–E (Pakistan).

***B.punctata* (Fabricius)**

Fig. 12

*Cicadapunctata* Fabricius, 1775: 687.

*Balcluthapunctata*: Blocker 1967: 7; Knight 1987: 1188, figs 32–38; Webb and Vilbaste 1994: 64, figs 44–54; Chiang 1996: 64, fig. 2; Dai, Li and Chen 2004: 749 (China); Naveed and Zhang 2018e: 261, figs 1A–C, 2F–K (Pakistan).

***B.pararubrostriata* Rao & Ramakrishnan**

*Balcluthapararubrostriata* Rao & Ramakrishnan, 1990a (India): 106; Webb and Vilbaste 1994: 64, fig. 130; Naveed and Zhang 2018e: 262, figs 1D–G, 3A–G (Pakistan).

***B.rubrostriata* (Melichar)**

*Gnathodusrubrostriatus* Melichar, 1903: 208.

*Balclutharubrostriata*: Knight 1987: 1211, figs 160–166; Webb and Vilbaste 1994: 66, figs 123–129; Chiang 1996: 69, fig. 5; Dai, Li and Chen 2004: 749 (China).

***B.sujawalensis* Ahmed**

*Balcluthasujawalensis* Ahmed, 1986: 54, fig. 2 (Pakistan).

*Balcluthaknighti* Rao & Ramakrishnan, 1990a: 106, figs 1–8 (India). Synonymised by Webb and Vilbaste 1994: 67, figs 55–60.

***A.viridinervis* Matsumura**

*Balcluthaviridinervis* Matsumura, 1914: 166; Knight 1987: 1190, figs 46–51; Webb and Vilbaste 1994: 69, figs 75–82; Khatri and Webb 2010: 13 (Pakistan).

### ﻿Key to Pakistan species of *Balclutha* (male) modified from Naveed and Zhang (2018e)

**Table:** 

1	Crown, pronotum and forewings with orange red longitudinal bands	**2**
–	Crown, pronotum and forewings without orange red longitudinal bands; aedeagus with basal processes	**3**
2	Pygofer with branches of posteroventral appendages only slightly divergent, extended posterad; distal part of aedeagal shaft distinctly curved in lateral view	***B.rubrostriata***
–	Pygofer with branches of posteroventral appendages widely divergent, one extended dorsad, the other ventrad; distal part of aedeagal shaft straight in lateral view	***B.pararubrostriata***
3	Sordid brown with brown markings (Fig. 12); aedeagal shaft short, C-shaped, curved dorsally and anteriorly to near level of basal apodeme	***B.punctata***
–	Yellowish green; aedeagal shaft not extending to near level of basal apodeme	**4**
4	Aedeagus with three or more pairs of processes, shaft not curved basally	***B.incisa***
–	Aedeagus without ventral processes, shaft curved basally	**5**
5	Aedeagus with basal apodeme finger-like in lateral aspect, shaft slightly sinuate apically	***B.viridinervis***
–	Aedeagus with basal apodeme not finger-like in lateral aspect, shaft not sinuate apically	***B.sujawalensis***

#### *Cicadulina* China

***C.bipunctata* (Melichar)**

*Gnathodusbipunctata* Melichar, 1904: 47.

*Cicadulabipunctella* Matsumura, 1914: 173 (Taiwan).

*Cicadulinabipunctata*: Webb 1987a: 236; Webb 1987b: 694, figs 70–77; Naveed and Zhang 2018e: 269, fig. 8A–E (Pakistan).

***C.chinai* Ghauri**

*Cicadulinachinai* Ghauri, 1964: 205 (India).

*Cicadulinastriata* Ahmed, 1986: 57, fig. 4, syn. nov.

*Cicadulinachinai*: Naveed and Zhang 2018e: 269, figs 7A–C, 8F–M (Pakistan).

**Remarks.** Original figures of *C.striata* show similarity to *C.chinai* in the shape of the pygofer process and aedeagus in lateral view but the aedeagus in posterior view (if drawn correctly) is a bit narrower. Described from the holotype male and several paratypes from Gharo, Thatta district, Sindh province, Pakistan maize, 11.x.85, Ahmed (ZMUK); no type specimens could be found.

### ﻿Key to Pakistan species of *Cicadulina* (male) modified from Naveed and Zhang 2018e)

**Table:** 

1	Pygofer with slender, hook-like process ending in triangular apex	***C.bipunctata***
6	Pygofer with thick and sinuate process, bifurcate at apex	***C.chinai***

#### *Macrosteles* Fieber

***M.indrina* (Pruthi)**

Figs 29, 64

*Cicadulaindrina* Pruthi, 1930: 61–62, pl. V fig. 5, text figs 83–84. N (India).

*Macrostelesindrina.* New combintion by Khatri and Webb 2010: 14, fig. 17.

*Macrostelesparafalcatus* Naveed & Zhang, 2018e: 266, figs 5A–J, 6A–C (Pakistan), syn. nov.

**Remarks.** A re-examination of the material identified and figured as *M.indrina* by Khatri and Webb (2010) and original figures of *M.parafalcatus* shows that there is insufficient evidence to separate the two species. The two species differ only very slightly in the separation of the long apodemes of the second abdominal sternite (fig. 64). Other differences seen in their respective original figures, i.e., of the aedeagus and style, are due to differences of orientation. Therefore, we consider the two species to be synonyms.

***M.shahidi* Ahmad**

*Macrostelesshahidi* Ahmed, 1986: 55, fig. 3 (Pakistan).

**Remarks.** The identity of this species is uncertain (see Khatri & Webb 2010: 14).

#### Mukariini Distant

**Diagnosis.** These are small to medium sized, often dorsoventrally depressed or ventrally flattened, brown, black, whitish, yellow, or green, leafhoppers, sometimes marked with orange or red. They can be identified by the produced head, often with frontoclypeus tumid distally, ventral part of face flat, lying nearly horizontally or concave, and ocelli distant from eyes.

#### *Mukaria* Distant

***M.splendida* Distant**

*Mukariasplendida* Distant, 1908: 270 (India); Khatri and Webb 2011: 19, figs 3a–k (Pakistan); Viraktamath and Webb 2019, figs 3A–D, 5R–S, 7D, 10A–D, 13E–I, 27A–J (India).

#### Opsiini Emaljanov

**Diagnosis.**Opsiini are small to large, stramineous, yellow, green, or brown leafhoppers. They can be identified by the bifurcate aedeagus with two shafts and gonopores. Some Mukariini and *Ascius* (Scaphytopiini) have a similarly divided aedeagus but Opsiini lack the other characters that define those groups.

#### *Hishimonus* Ishihara

***H.phycitis* (Distant)**

Figs 9, 44

*Eutettixphycitis* Distant, 1908: 363–364, fig. 231 (India).

*Eutettixlugubris* Distant, 1918: 60. Synonymised by Knight 1970: 128.

*Hishimonusorientalis* Emeljanov, 1969: 1102. Synonymised by Knight 1970: 128.

*Hishimonusphycitis*: Knight, 1970: 128–130, figs 10, 11, 13; Viraktamath and Murthy 2014: 114, figs 23–26, 161–176; Naveed and Zhang 2018j: 1805, figs 1A–B, 2A–J (Pakistan).

#### *Masiripius* Dlabola

***M.lugubris* (Distant)**

*Mahalanalugubris* Distant, 1918: 64 (India).

*Ziziphoidespunctatus*: Rao, 1967: 239, figs 1–6.

*Masiripiuslugubris*: Webb and Godoy 1993: 424; Viraktamath and Murthy 1999: 44, 47, figs 27–39 (India).

#### *Neoaliturus* Distant

**N. (Circulifer) tenellus (Baker)**

*Thamnotettixtenella* Baker, 1896: 24.

*Eutettixtenellus*: Uzel 1911: 287.

*Circulifertenellusambiguosus* Young & Frazier, 1954: 34, fig. 3.

*Neoaliturustenellus*: Nast 1972: 331.

Neoaliturus (Circulifer) tenellus Mozaffarian & Wilson, 2016: 24 (Iran).

**N. (Circulifer) opacipennis (Lethierry)**

*Cicadulaopacipennis* Lethierry, 1876: 83.

*Cicadulavittiventris* Lethierry, 1876: 84.

*Cicadulanausharensis* Pruthi, 1936: 113–114, fig. 127, pl. VIII, fig. 15 (Pakistan). Synonymised by Bindra et al. 1970: 664, figs 1–11.

*Neoaliturusopacipennis*: Mozaffarian and Wilson 2016: 24 (Iran).

### ﻿Key to Pakistan species of *Neoaliturus* (male)

**Table:** 

1	Subgenital plates widely truncated (Fig. 69)	**N. (C.) tenellus**
–	Subgenital plates acuminate (Fig. 70)	**N. (C.) opacipennis**

#### *Opsius* Fieber

***O.smaragdinus* (Distant)**

*Eutettixsmaragdinus* Distant, 1908: 364 (India).

*Cestiustriradiatus* Ahmed & Sultana, 1994: 129, fig. 2 (Pakistan).

*Opsiussmaragdinus*: Khatri and Webb 2010: 6.

***O.versicolor* (Distant)**

*Cestiusversicolor* Distant, 1908: 310, fig. 198 (India).

*Opsiusdissimilis* Vilbaste, 1961: 43.

*Cestiussakroensis* Ahmed & Sultana, 1994: 126, fig. 1 (Pakistan). Synonymised by Khatri and Webb 2010: 6.

*Opsiusversicolor*: El-Sonbati et al. 2020: 8, figs 13–18, 32–34, 47–49, 65–69.

### ﻿Key to Pakistan species of *Opsius* (male)

**Table:** 

1	Aedeagal shaft with ventral process directed away from aedeagal shaft dorsally	***O.versicolor***
–	Aedeagal shaft with ventral process close to aedeagal shaft dorsally	***O.smaragdinus***

#### *Orosius* Distant

***O.aegypticus* Ghauri**

Fig. 10

*Orosiusaegypticus* Ghauri, 1966: 251, fig. 11 (Egypt).

***O.albicinctus* Distant**

*Orosiusalbicinctus* Distant, 1918: 85 (India); Ghauri 1966: 236–239, fig. 3.

### ﻿Key to Pakistan species of *Orosius* (male)

**Table:** 

1	Aedeagal base bulbous	***O.aegypticus***
–	Aedeagal base not bulbous	***O.albicinctus***

#### Paralimnini Distant

**Diagnosis.** These are small to medium sized leafhoppers. They can be identified by the combination of the following characters: clypellus tapering apically or parallel-sided, lorum narrower than clypellus at base; connective with anterior arms closely appressed, articulated with aedeagus; female first valvula sculpturing imbricate or rarely maculate or granulose. The tribe is very similar morphologically to the closely related Deltocephalini, from which it can be distinguished by the articulation between the connective and aedeagus (fused in Deltocephalini), although a few species of *Flexamia* (Paralimnini) have the connective fused to the aedeagus.

**Remarks.**Khatri and Rustamani (2011) pointed out that the paralimnine *Hengchuniapakistanica*Asche and Webb (1994) was erroneously recorded from Pakistan as it is known from the Indian state of Gujarat (spelt as Gudjarat).

#### *Changwhania* Kwon

***C.ceylonensis* (Baker)**

*Deltocephalusbimaculatus* Melichar, 1903: 204 (Sri Lanka); Kuoh 1966: 128 (China).

*Deltocephalusceylonensis* Baker, 1925: 537. Replacement name for *Deltocephalusbimaculatus* Melichar.

*Cicadulabipunctatus* Pruthi, 1930:59, pl. V, fig. 3 (India). Synonymised by Webb and Heller 1990: 8.

*Changwhaniachangwhani* Kwon, 1980: 99, figs 1–8 (Korea). Synonymised by Webb and Heller 1990: 8.

*Changwhaniaceylonensis*: Webb and Heller 1990: 452; Zhang et al. 2009: 22 (China); Naveed and Zhang 2018f: 77, figs 1–14 (Pakistan).

***C.terauchii* (Matsumura)**

Fig. 18

*Aconuraterauchii* Matsumura, 1915: 163, Table 1, fig. 8; Matsumura 1931: 1250; Esaki and Ito 1954: 175.

*Changwhaniaterauchii* Kwon, 1980: 97–99, figs 1 (1–3), 2 (1–8) (Korea); Webb and Heller 1990: 452; Cai, Sun and Jiang 2001: 93; Zhang et al. 2009: 21 (China); Naveed and Zhang 2019b: 619, fig. 1 A–I (Pakistan).

### ﻿Key to species of *Changwhania* from Pakistan (male) modified from Naveed et al. (2019b)

**Table:** 

1	Crown with pair of round black anterior markings; aedeagus with subapical processes and truncate apex	***C.terauchii***
–	Crown with pair of oval black anterior markings; aedeagus with apical processes and apically rounded	***C.ceylonensis***

#### *Jilinga* Ghauri

***J.gopii* (Pruthi)**

*Deltocephalusgopii* Pruthi, 1936: 127, pl. IX, fig. 9, text fig. 138 (Pakistan).

*Jilingagopii* (Pruthi), comb. nov. by Webb & Heller, 1990: 8; Webb and Viraktamath 2009: 34; Khatri and Webb 2010: 15.

***J.neelumensis* Naveed & Zhang**

*Jilinganeelumensis* Naveed & Zhang, 2018g: 569, figs 1A–C, 3A–H, 4A–B (Pakistan).

***J.truncata* Naveed & Zhang**

Fig. 20

*Jilingatruncata* Naveed & Zhang, 2018g: 571, figs 1D–F, 2A–C, 5A–I (Pakistan).

### ﻿Key to *Jilinga* species of Pakistan (male) modified from Naveed and Zhang 2018g

**Table:** 

1	Anal tube ventral processes with fused section longer than distal branches, branches with only small denticuli present; aedeagal shaft broad in posterior view, no more than three times longer than wide	***J.gopii***
–	Anal tube ventral processes with fused section shorter than distal branches, branches with large teeth; aedeagal shaft narrow in posterior view, more than four times longer than wide	**2**
2	Dorsal connective less than twice as wide as distance between dorsal and ventral arms; anal tube appendage ventral branches with smaller teeth evenly distributed between pair of large teeth in posterior view	***J.neelumensis***
–	Dorsal connective more than twice as wide as distance between dorsal and ventral arms; anal tube appendage ventral branches with smaller teeth concentrated on large medial tooth	***J.truncata***

#### *Paralimnellus* Emeljanov

***P.cingulatus* (Dlabola)**

Figs 19, 35

*Paralimnuscingulatus* Dlabola, 1960: 2.

Paralimnus (Bubulcus) cingulatus Dlabola, 1961: 320.

*Paralimnelluscingulatus*: Emeljanov 1972: 107.

*Bubulcuscingulatus*: Hamilton 1975: 487; Webb and Heller 1990: 8.

Paralimnus (Dlabolasia) cingulatus: Nemesio 2007: 143.

*Paralimnelluscingulatus*: Xing and Li 2011: 54–56, figs 1–11 (China); Naveed and Zhang 2019b: 619, fig. 3A–J (Pakistan).

#### *Psammotettix* Haupt

***P.emarginata* Singh**

*Psammotettixemarginata* Singh, 1969: 356, figs 51–55 (India).

*Psammotettixswatensis* Ahmed, 1986: 52, fig. 1.

*Psammotettixquettensis* Ara & Ahmed, 1988: 292, fig. 2.

*Psammotettixemarginata*: Khatri and Webb 2010: 15, pl. 2f; figs 18, 19 (Pakistan).

#### *Soractellus* Evans

***S.nigrominutus* Evans**

Fig. 21

*Soractellusnigrominutus* Evans, 1966: 225–226, fig. 35H (Australia); Chalam and Subba Rao 2005: 234, figs 6–10 (India); Stiller 1988 (Africa); Xing and Li 2014: 298; Naveed and Zhang 2018k: 596 (Pakistan); Webb et al. 2019: 586, figs 1–5.

*Soractellusjianfengensis* Xing & Li, 2014: 297–300, figs 1–14, (China). Synonymised by Webb et al. 2019.

*Soractelluslalianensis* Naveed & Zhang, 2018k: 595–599 (Pakistan). Synonymised by Webb et al. 2019.

#### Penthimiini Kirschbaum

**Diagnosis.**Penthimiini are small to medium, squat, robust, often black or brown leafhoppers; often with ventral part of face and/or entire ventral side flattened and dorsal side convex. They can be identified by the ocelli on crown and often distant from eyes, strong antennal ledge, dorsally flattened and carinate protibia, and forewing with appendix large and extending around wing apex.

#### *Neodartus* Melichar

***N.acocephaloides* Melichar**

Fig. 2

*Neodartusacocephaloides* Melichar, 1903: 163; Distant 1908: 246, fig. 155; Distant 1918: 25; Rao 1993: 81–82 (India).

#### *Penthimia* Germar

***P.compacta* Walker**

*Penthimiacompacta* Walker, 1851: 842; Distant 1908: 242; Shobharani et al. 2018: 7, figs 5–9, 42, 56–60, 62, 69, 79–92, 172–175, 210–223 (India).

*Penthimiasubniger* Distant, 1908: 243–244, fig. 154.

*Penthimiascapularis* Distant, 1908: 244.

*Penthimiamaculosa* Distant, 1908: 244–245, in part.

#### Scaphoideini Oman

**Diagnosis.**Scaphoideini, following Zhaniser and Dietrich (2013: 148), is a rather poorly defined tribe. It was defined by these authors in the following way (with wording from their key to tribes in square brackets and added characters from Viraktamath and Yeshwanth (2020) in bold): “None of the following characters are present in all taxa, but some combination of [most of] these characters is present in all and a few (*) appear to be unique to this tribe: head narrower than pronotum, produced; **genae sometimes wide and visible dorsally**; frontoclypeus long and narrow; antennae long [longer than width of head]; body slender; head and wings often with brown, orange, ochraceous, or ivory markings; forewing with one or more darkly pigmented reflexed veins in vicinity of outer anteapical cell; profemur row AV setae absent or reduced (without stout setae); metatibia macrosetae in row PD long, as long as or longer than 0.5x length of protibia*; male or female pygofer with dense tufts of long fine or regular [macro] setae*; subgenital plate apex membranous or long, digitate, and somewhat membranous or weakly sclerotised; subgenital plates with long fine setae laterally and/or dorsally (also occurs in other deltocephaline tribes); basal processes of aedeagus or connective sometimes present, connected or articulated to base of aedeagus or apex of connective stem; **aedeagus sometimes fused to connective”.** The last mentioned character is found in *Sikhamani* Viraktamath and Webb and *Thryaksha* Viraktamath and Murthy.

#### *Bampurius* Dlabola

***B.pakistanicus* Khatri & Webb**

*Bampuriuspakistanicus* Khatri & Webb, 2010: 18, pl. 1a; figs 1, 2 (Pakistan).

#### *Grammacephalus* Haupt

***G.genoicus* Dlabola**

*Grammacephalusgenoicus* Dlabola, 1984: 52; Khatri and Webb 2010: 16, pl. 2g; fig. 22 (Pakistan).

***G.indicus* Viraktamath & Murthy**

*Grammacephalusindicus* Viraktamath & Anantha Murthy, 1999: 42 (india); Khatri and Webb 2010: 16, pl. 2h; figs 20–21; Naveed and Zhang 2018h: 1816, fig. 1A–I (Pakistan).

***G.pallidus* Linnavuori**

*Grammacephaluspallidus* Linnavuori, 1978: 479; Viraktamath 1981: 8, figs 10–17 (Indicus); Khatri and Webb 2010: 16, pl. 2i; fig. 23 (Pakistan).

***G.punjabensis* Shah & Duan**

*Grammacephaluspunjabensis* Shah & Duan, 2019: 82, figs 11, 12 (Pakistan).

***G.rahmani* (Pruthi)**

*Platymetopiusrahmani* Pruthi, 1930: 33, pl. III, figs 2, 2a, text figs 45–46 (Pakistan, India).

*Grammacephalusrahmani* (Pruthi, 1930: 33), Mahmood 1979; Viraktamath 1981: 7, figs 1–9; Khatri and Webb 2010: 16.

***G.raunoi* Viraktamath**

Figs 15, 33

*Grammacephalusraunoi* Viraktamath, 1981: 9, figs 30–36 (India); Naveed and Zhang 2018h: 1816, fig. 2A–J (Pakistan).

### ﻿Key to species of *Grammacephalus* from Pakistan (male) modified from Naveed and Zhang (2018h)

**Table:** 

1	Male pygofer process absent	***G.genoicus***
–	Male pygofer process present	**2**
2	Pygofer process with an appendage; aedeagal shaft with median expansion laterally	***G.raunoi***
–	Pygofer process without appendage; aedeagal shaft without median expansion laterally	**3**
3	Pygofer process with bifurcated apex	***G.punjabensis***
–	Pygofer process without bifurcated apex	**4**
4	Aedeagal shaft tubular	***G.rahmani***
–	Aedeagal shaft not tubular	**5**
5	Aedeagal shaft strongly reflexed basally, rather incrassate	***G.pallidus***
–	Aedeagal shaft not strongly reflexed basally, not incrassate	***G.indicus***

#### *Monobazus* Distant

***M.dissimilis* (Distant)**

*Xestocephalusdissimilis* Distant, 1918: 55 (India).

*Deltocephalusfuscovarius* Distant, 1918: 83. Synonymised by Webb and Viraktamth 2009: 29

*Monobazusdissimilis*: Khatri and Webb 2010: 7, pl. 1d; fig. 4 (Pakistan).

#### *Neolimnus* Linnavuori

***N.egyptiacus* (Matsumura)**

Fig. 16

*Scaphoideusegyptiacus* Matsumura, 1908: 29.

*Neolimnusegyptiacus* Linnavuori, 1953: 114; Khatri and Webb 2010: 7, pl. 1c; fig. 7.

*Scaphoideuskarachiensis*Ahmed et al., 1988: 410 (Pakistan). Synonymised by Khatri and Webb 2010: 7.

#### Osbornellus (Mavromoustaca) Dlabola

**O. (M.) macchiae Lindberg**

*Circulifermacchiae* Lindberg, 1948: 160.

Osbornellus (Mavromoustaca) consanguineus Dlabola, 1967: 38. Synonymised by Kartel 1982: 27.

Osbornellus (Mavromoustaca) macchiae Khatri & Webb, 2010: 8, pl. 1e; fig. 3 (Pakistan).

#### *Phlogotettix* Ribaut

***P.indicus* Rao**

Fig. 75

*Phlogotettixindicus* Rao, 1989: 77; Meshram et al. 2015: 234, figs 22–36 (India).

#### *Scaphoideus* Uhler

***S.harlani* Kitbamroong & Freytag**

Fig. 17, 55

*Scaphoideusharlani* Kitbamroong & Freytag, 1978: 11; Khatri and Webb 2010: 8, pl. 1f; fig. 8 (Pakistan).

#### Stenometopiini Baker

**Diagnosis.** These are small to medium sized, rarely brightly coloured but iridescent leafhoppers when alive. They can be identified by the narrow crown, shagreen texture of crown, clypellus parallel-sided or tapering apically, forewings often submacropterous to brachypterous, male pygofer sloping caudoventrally and with few macrosetae and often with a distinct lateral tooth, female ovipositor protruding far beyond the pygofer apex, first valvula dorsal sculpturing granulose to maculate and submarginal, first valvula with distinctly delimited ventroapical sculpturing, and second valvula without dorsal teeth.

#### *Stirellus* Osborn & Ball

***S.kumratensis* Naveed & Zhang**

*Stirelluskumratensis* Naveed & Zhang, 2020b: 481, figs 5, 6, 9–15 (Pakistan).

***S.lahorensis* (Distant)**

Fig. 54

*Volusenuslahorensis* Distant, 1918: 72 (Pakistan).

*Stirelluspeshawarensis* Mahmood, Sultana & Waheed, 1972: 80. Synonymised by Khatri and Webb 2010.

*Paternusjhokensis* Ahmed & Aziz, 1988: 805. Synonymised by Khatri and Webb 2010.

*Stirelluslahorensis*: Khatri and Webb 2010: 17, pl. 2j; fig. 24; Naveed and Zhang 2020b: 480, figs 1, 2 (Pakistan).

***S.mankiensis* Shah & Duan**

Figs 24, 32

*Stirellusmankiensis* Shah & Duan, 2020a: 198, figs 9, 10 (Pakistan).

***S.neoconvexus* Naveed & Zhang**

*Stirellusneoconvexus* Naveed & Zhang, 2020b: 481, figs 7, 8, 16–20 (Pakistan).

***S.thattaensis* Mahmood, Sultana & Waheed**

Fig. 63

*Stirellusthattaensis* Mahmood, Sultana & Waheed, 1972: 82, fig. 2 (Pakistan).

***S.viridulus* (Pruthi)**

Fig. 71

*Paternusviridula* Pruthi, 1930: 42, pl. IV, figs 1, 1a, text figs 57–59 (India).

*Paternusviridulus* Metcalf, 1967a: 2350.

*Stirellusviridulus*: Khatri and Webb 2010: 1–47; Naveed and Zhang 2020b: 481, figs 3, 4 (Pakistan).

***S.tolla* (Pruthi)**

*Aconuratolla* Pruthi, 1930: 39, pl. III, figs 7, 7a, text fig. 54 (India); Shah and Duan 2020a: 196, figs 6–8 (Pakistan).

### ﻿Key to species of the genus *Stirellus* from Pakistan (male) modified from Shah et al. (2020)

**Table:** 

1	Crown 1.5 × longer than breadth between eyes	***S.lahorensis***
–	Crown less than 1.5 × or equal to breadth between eyes	**2**
2	Species yellowish green in colour	**3**
–	Species ochraceous to brownish in colour	**5**
3	Crown anterior margin very slightly angulate	***S.tolla***
–	Crown anterior margin acutely angled	**4**
4	Male pygofer long, with rounded apex (Fig. 71)	***S.viridulus***
–	Male pygofer short with pointed apex (Fig. 63)	***S.thattaensis***
5	Subgenital plate with macrosetae uniseriate laterally	***S.kumratensis***
–	Subgenital plate with macrosetae not uniseriate laterally	**6**
6	Connective stem shorter than anterior arms, aedeagal shaft with blunt apex	***S.neoconvexus***
–	Connective stem longer than anterior arms, aedeagal shaft with pointed apex	***S.mankiensis***

#### Vartini Zahniser & Dietrich

**Diagnosis.**Vartini are medium sized to large, somewhat elongate, greenish or bluish leafhoppers, usually with red or orange longitudinal stripes. They can be identified by the produced and pointed head, gena visible behind eye in dorsal view, elongate frontoclypeus, lorum distant from genal margin, profemur intercalary row setae thick and extending to or beyond middle of profemur, forewings truncate apically, apodemes of male sternite II long, subrectangular, flared apically, and pointed posterolaterally, connective with anterior arms appressed, and male segment X tube-like and protruding from pygofer and often well sclerotised.

#### *Varta* Distant

***V.rubrofasciata* Distant**

*Vartarubrofasciata* Distant, 1908: 321, fig. 205 (India); Viraktamath 2004: 13, figs 33, 49, 50 (India, Taiwan).
